# Exposure–response analyses of erdafitinib in patients with locally advanced or metastatic urothelial carcinoma

**DOI:** 10.1007/s00280-021-04381-4

**Published:** 2022-01-03

**Authors:** Anne-Gaëlle Dosne, Elodie Valade, Nele Goeyvaerts, Peter De Porre, Anjali Avadhani, Anne O’Hagan, Lilian Y. Li, Daniele Ouellet, Juan Jose Perez Ruixo

**Affiliations:** 1 Janssen Research and Development, Beerse, Belgium; 2 Janssen Research and Development, Spring House, PA USA

**Keywords:** Erdafitinib, Exposure–response analyses, FGFR inhibitor, Metastatic urothelial carcinoma, Pharmacodynamically guided individual dose titration

## Abstract

**Background:**

Exposure–response analyses were conducted to explore the relationship between selected efficacy and safety endpoints and serum phosphate (PO4) concentrations, a potential biomarker of efficacy and safety, in locally advanced or metastatic urothelial carcinoma patients with FGFR alterations treated with erdafitinib.

**Methods:**

Data from two dosing regimens of erdafitinib in a phase 2 study (NCT02365597), 6 and 8-mg/day with provision for pharmacodynamically guided titration per serum PO4 levels, were analyzed using Cox proportional hazard or logistic regression models. Efficacy endpoints were overall survival (OS), progression-free survival (PFS), and objective response rate (ORR). Safety endpoints were adverse events typical for FGFR inhibitors.

**Results:**

Exposure-efficacy analyses on 156 patients (6-mg = 68; 8-mg = 88) showed that patients with higher serum PO4 levels within the first 6 weeks showed better OS (hazard ratio 0.57 [95% CI 0.46–0.72] per mg/dL of PO4; *p* = 0.01), PFS (hazard ratio 0.80 [0.67–0.94] per mg/dL of PO4; *p* = 0.01), and ORR (odds ratio 1.38 [1.02–1.86] per mg/dL of PO4; *p* = 0.04). Exposure-safety analyses on 177 patients (6-mg = 78; 8-mg = 99) showed that the incidence of selected adverse events associated with on-target off-tumor effects significantly rose with higher PO4.

**Conclusions:**

The exploratory relationship between serum PO4 levels and efficacy/safety outcomes supported the use of pharmacodynamically guided dose titration to optimize erdafitinib’s therapeutic benefit/risk ratio.

**Clinical trial registration number:**

NCT02365597.

**Supplementary Information:**

The online version contains supplementary material available at 10.1007/s00280-021-04381-4.

## Introduction

Precision medicine, i.e. identifying and targeting specific molecular alterations involved in disease pathophysiology, is rapidly evolving in oncology [1]. Genomic alterations in the fibroblast growth factor receptor (FGFR) have been widely described in patients with urothelial carcinoma and led to extensive studies on treatment approaches with FGFR inhibitors [2, 3]. Approximately, 15–20% of patients with advanced or metastatic urothelial carcinoma (mUC) have been reported to have FGFR alterations [4]. Erdafitinib, a once-daily oral FGFR tyrosine kinase inhibitor, was recently approved by the US FDA for patients with locally advanced or mUC, with susceptible FGFR3 or FGFR2 genetic alterations that has progressed during or following at least one line of prior platinum-containing chemotherapy, including within 12 months of neoadjuvant or adjuvant platinum-containing chemotherapy [5].

Due to the blockade of renal FGF23 signaling through FGFR tyrosine kinase inhibition, an increase in serum phosphate (PO4) concentration was observed after dosing [6–8]. This elevation of serum PO4 is a known class effect of FGFR inhibitors [9, 10], and serum PO4 was considered as a pharmacodynamic (PD) marker of FGFR engagement and proposed to be a biomarker of efficacy and safety [11].

The phase 1 data (EDI1001; NCT01703481) of erdafitinib coupled with pharmacokinetic (PK)/PD modeling identified a serum PO4 level of ≥ 5.5 mg/dL as PD target with acceptable tolerability and 7 mg/dL PO4 was considered the threshold for dosing adjustments [11]. To maximize efficacy while limiting its toxicity, a pharmacodynamically guided individual dose titration for erdafitinib was proposed and evaluated in the pivotal Phase 2 study (BLC2001; NCT02365597) [12].

Understanding the relationship between serum PO4 concentration and response/safety is important to optimize dosing regimens and assess the benefit-risk profile. The relationships between erdafitinib doses and erdafitinib plasma concentrations [13], as well as between erdafitinib plasma concentrations and serum PO4 concentrations [14], have been characterized using a population PK-PD model. In this study, using data from study BLC2001 [12], we performed exposure–response (ER) analyses that explored the relationships between serum PO4 concentrations and selected clinical endpoints of efficacy and safety in patients with locally advanced or mUC with certain FGFR genetic alterations. The goal is to evaluate serum PO4 as a biomarker for erdafitinib dose individualization and to support the approved pharmacodynamically guided individual dose titration including up-titration and dose reduction strategies.

## Methods

### Analysis data

Data up to May 2018 were collected from the two once-daily dosing regimen arms of phase 2, multicenter, open-label study BLC2001. Study design and primary outcomes of BLC2001 were discussed in a separate publication [12]. The primary endpoint of the study was the objective response rate (ORR) with key secondary endpoints including progression-free survival (PFS), and overall survival (OS). The first continuous regimen (Regimen 1) was a 6 mg daily dose, with possible up-titration to 8 mg at the end of Cycle 1 (C1D28) if PO4 concentration was < 5.5 mg/dL and no significant toxicity.

The second continuous regimen (Regimen 2) was a 8 mg daily dose with possible up-titration to 9 mg if the C1D14 PO4 concentration was < 5.5 mg/dL and in the absence of significant toxicity [12]. Data from the third, intermittent regimen were not included in this analysis due to the insufficient clinical benefit observed for this regimen.

### Efficacy endpoints

Data used in the efficacy analysis included all chemotherapy relapsed or refractory patients in Regimen 1 and Regimen 2 who received at least 1 dose of the study drug. Efficacy endpoints included OS, PFS, and ORR (for definitions: see Appendix). Patients were assessed for disease response every 6 weeks during the first 3 months, every 12 weeks for the next 9 months, and thereafter every 4–6 months until disease progression. Erdafitinib treatment was to be discontinued at the time of disease progression or unacceptable toxicity as determined by the investigator. Patients who had investigator-assessed disease progression could continue to receive erdafitinib based on sponsor and treating physician agreement for perceived clinical benefit. After discontinuation of erdafitinib, patients were assessed every 12 weeks for survival status.

### Safety endpoints

Data used in the safety analysis included all patients in Regimen 1 and Regimen 2 who received at least 1 dose of the study drug. The safety endpoints were selected based on their clinical relevance, incidence (> 10%), or presence of grade ≥ 3 severity. Key safety endpoints included eye disorders, central serous retinopathy (CSR), nail disorders, palmar-plantar erythrodysesthesia syndrome (PPES), and skin disorders (Appendix). The selected safety endpoints were dichotomized into the presence or absence of such AEs. The dichotomization was done irrespective of severity grade due to the limited number of events with grade 3 or grade 4, which was too low to enable a severity-based analysis. Patients with multiple occurrences were only counted once, at the time when the highest severity was first experienced.

### Exposure metrics

Serum PO4 concentration was postulated to be a biomarker of tumor response since it reflects tumor FGFR engagement as evidenced by FGFR mediated effects in the kidneys [6, 7, 15]. It was also postulated to be a biomarker of safety endpoints. Area under the curve of erdafitinib plasma concentration over time (AUC) was also evaluated as a biomarker of efficacy and safety endpoints. Serum PO4 and erdafitinib AUC were derived from previously developed population PK and PK-PD models [13], which were fitted to the available data to derive individual PK and PD parameters for every patient.

For efficacy endpoints (OS, PFS and ORR), the exposure metric obtained for each patient was the PK-PD model[13]-predicted average daily serum PO4 concentration up to the day of first response assessment (i.e., 6 weeks per the study protocol), PO4_ave,6 weeks_. In addition, for the OS and PFS analyses, average daily serum PO4 concentration was also computed for each week to investigate whether taking into account PO4 changes over time in a more granular manner better-predicted efficacy. For safety endpoints, the exposure metric obtained for each patient was the model-predicted average daily serum PO4 concentration up to the day of AE (PO4_ave,event_). For efficacy and safety analyses, early PO4 metrics (average daily PO4 concentration up to 14 days, PO4_ave,2 weeks_) were explored to investigate how PO4 concentrations before up-titration correlated with the different endpoints. Average daily plasma AUC (AUC_ave,6 weeks_ and AUC_ave,event_) were also explored to investigate whether erdafitinib PK correlated better with the different endpoints than serum PO4. Other PK metrics (minimum and maximum concentrations *C*_min_ and *C*_max_) were also evaluated, but are not reported here as they yielded identical results to AUC due to the high correlation observed between the metrics. Both free and total AUC were investigated as erdafitinib is highly bound to plasma proteins (> 99%), which is variable between patients. Free concentrations are physiologically expected to correlate with a drug effect. Free fraction was determined from the plasma protein-binding sample using equilibrium dialysis [16].

### Prognostic factors for exposure-efficacy analysis

The prognostic factors included in the exposure-efficacy analysis were ECOG performance status (> 1 vs ≤ 1), hemoglobin level (≤ 10 vs > 10 g/L), presence of liver, bone or lung metastases and FGFR alteration type (fusion versus mutation) (Appendix). Prognostic and predictive factors were not included in the exposure-safety analyses as none of the evaluated factors were found significant by statistical analysis.

### Statistical methods

All ER analyses were performed on the combined data of the continuous dose regimens. These data could be combined as differences in titration schemes (dose levels and timing of up-titration) were directly reflected in the metrics used for analysis (PO4 and AUC). By taking the average PO4 or AUC until a given event (first response assessment for efficacy or first event of the highest grade for safety), each patient’s individual dosing history and drug sensitivity were taken into account and patients could be analyzed across regimens. Results of the ER analyses were expressed as hazard ratio (HR) for OS and PFS or odds ratio (OR) for ORR. More details on the statistical analysis methods can be found in Appendix.

#### Exposure-efficacy analysis

Exploratory Kaplan–Meier analyses were conducted for OS and PFS stratified by PO4 concentration (three groups of low, medium, and high PO4 concentration based on PO4 terciles). The ER analyses for these endpoints were performed using univariate and multivariate Cox regression models including serum PO4 concentration as well as selected prognostic factors as covariates. Corresponding HR, 95% confidence intervals (CI), and *p* values were obtained. In addition, time-dependent PO4 analyses, assuming a direct effect of weekly serum PO4 concentrations while on treatment on the OS or PFS hazard, were performed to further investigate how variations of PO4 over time could affect the risk of disease progression and/or death.

For the ORR, univariate and multivariate logistic regression models were used to estimate the relationship between serum PO4 concentrations as well as selected prognostic factors and ORR. Corresponding OR, 95% CI, and *p* values were obtained.

Plasma erdafitinib AUC was also investigated as biomarker for all efficacy endpoints. For all analyses, the selection of the final model was based on the value of the log-likelihood together with the number of model parameters (if the models were nested, i.e., if one model contained all the terms of the others and at least one additional term) or Akaike’s Information Criterion[17] (if the models were not nested), the magnitude of the HR or OR and its associated statistical significance, and the consistency of the effect between univariate and multivariate models.

#### Exposure-safety analysis

Univariate logistic regression models were used to assess the relationship between serum PO4 concentrations and the incidence of selected treatment-emergent adverse events (TEAEs). Plasma erdaftinib AUC was also investigated as biomarker for all safety endpoints.

## Results

A total of 210 patients were enrolled in the study BLC2001 at the time of data cut-off, of which 177 received the continuous dose regimens (safety analysis dataset). Of these 177 patients, 156 were chemotherapy relapsed or refractory patients in Regimen 1 and Regimen 2 (efficacy analysis dataset). A summary of the demographic and disease characteristics of the patients at baseline are listed in Table 1 (safety population) and Table S1 (efficacy population). Regarding prognostic factors, most patients had lung metastases, FGFR mutations, low ECOG score and high hemoglobin levels. The sample size to assess the effect of the different prognostic factors was limited, with between 12 and 35 patients in the least frequent groups. The mean baseline serum PO4 concentration was 3.44 mg/dL. Phosphate-lowering drugs were taken by 39 patients (*n* = 5 took denosumab, *n* = 3 took zoledronic acid and *n* = 31 took phosphate binders). The adequacy of the PK-PD model to describe the erdafitinib concentrations and PO4 concentrations from the study BLC2001 was confirmed (Figs S1 and S2).

**Table 1:** Summary of demographic and disease characteristics of the patients at baseline in the exposure–response analysis dataset

Characteristics	Exposure–response dataset (safety^a^, *n* = 177)
Age (years), Mean (SD)	65.4 (10.33)
Men, *n* (%)	130 (73.4)
Body weight (kg), Mean (SD)	74.7 (19.06)
AGP (g/L), Mean (SD)	1.33 (0.618)
Free fraction^b^ (%), Mean (SD)	0.28 (0.175)
Race, *n* (%)	
White	125 (70.6)
Black	4 (2.3)
Asian	16 (9.0)
Other	32 (18.1)
Renal impairment, *n* (%)	
Normal	23 (13.0)
Mild	64 (36.2)
Moderate	90 (50.8)
ECOG^c^, *n* (%)	
Grade 0	65 (41.7)
Grade 1	79 (50.6)
Grade 2	12 (7.7)
Disease distribution^c^, *n* (%)	
Absence of visceral metastases	32 (20.5)
Presence of visceral metastases	124 (79.5)
Liver	40 (22.6)
Bone	32 (18.1)
Lung	86 (48.6)
FGFR alteration type^c^, *n* (%)	
Mutation	126 (80.8)
Fusion	30 (19.2)
Hemoglobin^c^ (g/dL), Mean (SD)	11.8 (1.88)
Prior or concomitant phosphate modifying medications, *n* (%)	39 (22.0)
Denosumab	5 (2.8)
Zoledronate	3 (1.7)
Phosphate binders	31 (17.5)
Baseline phosphate concentration^d^ (mg/dL), Mean (SD)	3.44 (0.56)

*AGP* alpha-1-acid glycoprotein; *ECOG* Eastern Cooperative Oncology Group; *FGFR* fibroblast growth factor receptor; *SD* standard deviation

^a^Characteristics similar for the efficacy analysis dataset, which is a subset of the safety dataset (*n* = 156)

^b^Free fraction of erdafitinib was derived from total and free erdafitinib concentration measurements

^c^ECOG, disease distribution, FGFR alteration type, and hemoglobin were computed based on the efficacy dataset as these variables are prognostic factors (*n* = 156)

^d^Baseline phosphate missing for 88 out of 177 patients (50%)

### Exposure-efficacy analyses

For the efficacy analyses, data from 156 patients in the erdafitinib 8 mg (*n* = 88) or 6 mg (*n* = 68) once-daily regimens were included. The average PO4 concentrations derived from the PK-PD model[13] are provided in Table S2 for the pooled analysis and by dose regimen.

#### ER efficacy: OS

**Fig. 1:**
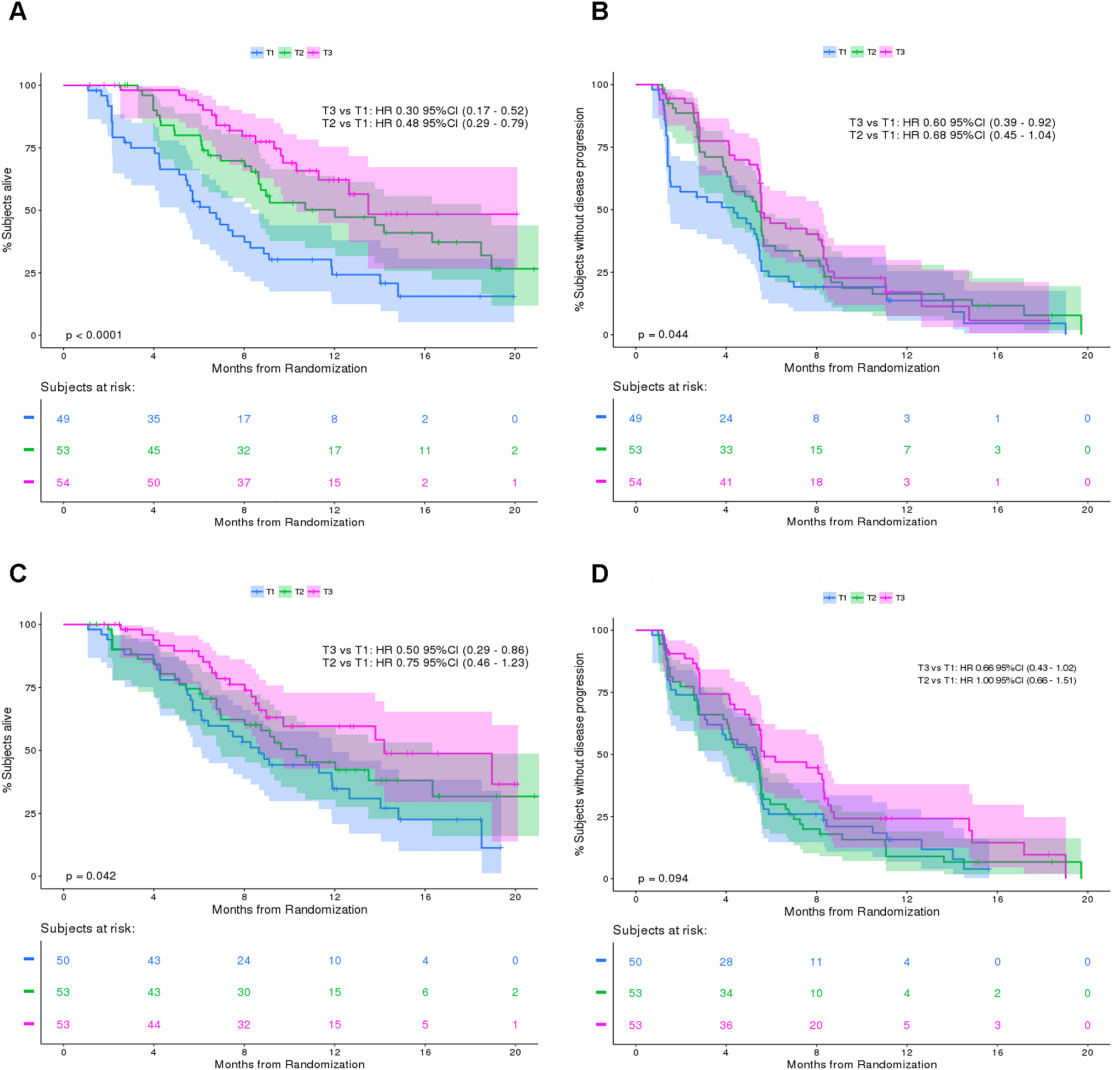
Kaplan–Meier plot for **A** overall survival and **B** progression-free survival for terciles of average daily phosphate exposure up to 6 weeks **C** overall survival and **D** progression-free survival for terciles of average free erdafitinib exposure up to 6 weeks. T1, T2, and T3 represent the lowest (2.4–4.8 mg/dL), middle (4.8–5.6 mg/dL), and highest (5.6–10.4 mg/dL) terciles of serum phosphate concentrations in panels **A** and **B** and the lowest (10.6–51.2 ng.h/mL), middle (51.6–74.7 ng.h/mL) and highest (75.5–307 ng.h/mL) terciles of erdafitinib free AUC in panels *C* and **D**. *CI* confidence interval; *HR* hazard ratio; *PFS* progression-free survival

Median OS was 10.7 (95% CI 8.6, 13.8) months in the efficacy analysis dataset, with 26% of patients still alive at 12 months. When stratifying by dose regimen, the median OS was 13.8 months in the 8 mg regimen versus 8.9 months in the 6 mg regimen. When stratifying by erdafitinib free AUC, patients with higher AUC (highest tercile) showed a median OS of 14.2 months compared to 10.3 months in patients with medium AUC (mid tercile) and 8.6 months in patients with lower AUC (lowest tercile, Fig. 1C). Stratifying by serum PO4 (PO4_ave,6 weeks_) showed the greatest OS differences. Patients with higher serum PO4 level (highest tercile) showed a median OS of 13.5 months compared to 12.0 months in patients with medium PO4 level (mid tercile) and 6.5 months in patients with lower serum PO4 level (lowest tercile). The ER analysis revealed a statistically significant relationship between serum PO4 level and OS, both when serum PO4 concentration was categorized by terciles (*p* < 0.001, Fig. 1A) and when it was used as a continuous variable (HR 0.61; 95% CI 0.48, 0.77 per 1 mg/dL increase in average daily serum PO4 concentration; *p* < 0.001, final model, Table 2).

**Table 2:** Results of exposure–response analyses

Parameter	− 2LL^a^	AIC^a^	Biomarker/Prognostic factor	HR/OR (95% CI)^b^	*p* value^c^
Efficacy endpoints					
OS			Univariate analysis		
	710.5	712.5	PO4_ave,6 weeks_ (per 1 mg/dL increase)	0.57 (0.46–0.72)	< 0.001
	729.8	731.8	Hemoglobin (≤ 10 vs > 10 g/L)	1.72 (1.05–2.80)	0.03
	729.6	731.6	ECOG (> 1 vs ≤ 1)	2.32 (1.15–4.65)	0.02
	720.8	722.8	Liver metastases (yes vs no)	2.50 (1.57–3.98)	< 0.001
	729.1	731.1	Bone metastases (yes vs no)	1.83 (1.11–3.03)	0.02
	734.2	736.2	Lung metastases (yes vs no)	0.99 (0.64–1.53)	0.96
	733.9	735.9	FGFR alteration (fusion vs mutation)	0.93 (0.70–1.23)	0.59
	700.6	714.6	Full model		
			PO4_ave,6 weeks_ (per 1 mg/dL increase)	0.63 (0.49–0.80)	< 0.001
			Hemoglobin (≤ 10 vs > 10 g/L)	1.10 (0.62–1.91)	0.76
			ECOG (> 1 vs ≤ 1)	1.49 (0.70–3.16)	0.3
			Liver metastases (yes vs no)	1.73 (0.99–3.04)	0.36
			Bone metastases (yes vs no)	1.31 (0.74–2.32)	0.05
			Lung metastases (yes vs no)	0.87 (0.55–1.33)	0.49
			FGFR alteration (fusion vs mutation)	1.04 (0.77–1.40)	0.81
	703.2	707.2	Final model		
			PO4_ave,6 weeks_ (per 1 mg/dL increase)	0.61 (0.48–0.77)	< 0.001
			Liver metastases (yes vs no)	1.97 (1.22–3.16)	0.005
PFS			Univariate analysis		
	1103.2	1105.2	PO4_ave,6 weeks_ (per 1 mg/dL increase)	0.80 (0.67–0.95)	0.01
	1109.9	1111.9	Hemoglobin (≤ 10 vs > 10 g/L)	1.05 (0.69–1.59)	0.83
	1106.0	1108.0	ECOG (> 1 vs ≤ 1)	1.97 (1.07–3.64)	0.03
	1101.7	1103.7	Liver metastases (yes vs no)	1.81 (1.23–2.67)	0.003
	1107.8	1109.8	Bone metastases (yes vs no)	1.38 (0.90–2.11)	0.14
	1109.6	1111.6	Lung metastases (yes vs no)	0.90 (0.64–1.28)	0.57
	1110.0	1112.0	FGFR alteration (fusion vs mutation)	1.01 (0.81–1.25)	0.93
	1092.4	1106.4	Full model		
			PO4_ave,6 weeks_ (per 1 mg/dL increase)	0.84 (0.70–1.01)	0.07
			Hemoglobin (≤ 10 vs > 10 g/L)	0.74 (0.46–1.18)	0.21
			ECOG (> 1 vs ≤ 1)	1.69 (0.88–3.27)	0.12
			Bone metastases (yes vs no)	1.15 (0.73–1.83)	0.54
			Liver metastases (yes vs no)	1.80 (1.13–2.85)	0.01
			Lung metastases (yes vs no)	0.84 (0.59–1.21)	0.35
			FGFR alteration (fusion vs mutation)	1.10 (0.87–1.38)	0.43
	1097.9	1101.9	Final model		
			PO4_ave,6 weeks_ (per 1 mg/dL increase)	0.84 (0.70–1.00)	0.05
			Liver metastases (yes vs no)	1.63 (1.09–2.46)	0.02
ORR			Univariate analysis		
	207.3	211.3	PO4_ave,6 weeks_ (per 1 mg/dL increase)	1.29 (0.98–1.74)	0.08
	209.7	213.8	Hemoglobin (≤ 10 vs > 10 g/L)	0.72 (0.32–1.55)	0.40
	213.1	209.1	ECOG (> 1 vs ≤ 1)	0.47 (0.10–1.64)	0.27
	209.0	213.0	Liver metastases (yes vs no)	0.64 (0.29–1.34)	0.24
	210.3	214.3	Bone metastases (yes vs no)	0.86 (0.38–1.89)	0.71
	209.9	213.9	Lung metastases (yes vs no)	1.28 (0.67–2.45)	0.46
	203.6	207.6	FGFR alteration (fusion vs mutation)	0.55 (0.33–0.87)	0.015
	195.4	211.4	Full model		
			PO4_ave,6 weeks_ (per 1 mg/dL increase)	1.34 (0.98–1.84)	0.07
			Hemoglobin (≤ 10 vs > 10 g/L)	0.92 (0.38–2.17)	0.85
			ECOG (> 1 vs ≤ 1)	0.44 (0.09–1.67)	0.26
			Bone metastases (yes vs no)	0.84 (0.35–1.96)	0.70
			Liver metastases (yes vs no)	0.74 (0.31–1.72)	0.49
			Lung metastases (yes vs no)	1.40 (0.71–2.80)	0.33
			FGFR alteration (fusion vs mutation)	0.48 (0.28–0.77)	0.004
	198.7	206.7	Interaction model		
			PO4_ave,6 weeks_ (per 1 mg/dL increase)	1.56 (0.86–2.90)	0.15
			FGFR alteration (fusion vs mutation)	0.81 (0.09–6.37)	0.85
			Interaction (PO4 for fusion)^d^	0.92 (0.64–1.33)	0.65
	198.9	204.9	Final model		
			PO4_ave,6 weeks_ (per 1 mg/dL increase)	1.38 (1.02–1.86)	0.04
			FGFR alteration (fusion vs mutation)	0.26 (0.10–0.70)	0.01
Safety endpoints (univariate analyses)					
Nail disorders	213.3	217.3	PO4_ave,event_ (per 1 mg/dL increase)	2.84 (1.87–4.31)	< 0.001
Eye disorders	217.3	221.3	PO4_ave,event_ (per 1 mg/dL increase)	2.44 (1.65–3.62)	< 0.001
Skin disorders	222.5	226.5	PO4_ave,event_ (per 1 mg/dL increase)	1.61 (1.14–2.27)	0.007
PPES	171.7	175.7	PO4_ave,event_ (per 1 mg/dL increase)	1.72 (1.15–2.59)	0.009
CSR	159.7	163.7	PO4_ave,event_ (per 1 mg/dL increase)	1.97 (1.30–3.00)	0.002

*− 2LL* − 2 log-likelihood; *AIC* Akaike’s Information Criterion; *CI* confidence interval; *CSR* central serous retinopathy; *HR* hazard ratio; *OR* odds ratio; *ORR* objective response rate; *PFS* progression-free survival; *PPES* palmar-plantar erythrodysesthesia syndrome; *PO4*_*ave,6 weeks*_ average daily serum phosphate until the first response assessment; *PO4*_*ave,event*_ average daily serum phosphate until the first highest grade adverse event

^a^Best model has significantly lower − 2LL (nested models) or lowest AIC (non-nested models)

^b^HR for OS and PFS, OR for ORR and safety endpoints

^c^*p* values are rounded to 2 decimals and have 0.001 as lower bound

^d^Equivalent OR PO4_ave,6 weeks_ (per 1 mg/dL increase) for fusion: 1.43; OR for fusion vs mutation given mean PO4_ave,6 weeks_ = 5.30 mg/dL: 0.52

When testing the effect of the prognostic factors in univariate analyses, high ECOG, low hemoglobin, liver and bone metastases were found statistically significant adverse prognostic factors (Table 2). When adding PO4 in the full model (Table 2), the HRs related to the prognostic factors decreased and none of the prognostic factors remained statistically significant, liver metastases being at the border of an effect (HR [95% CI] 1.73 [0.99–3.04]). As the estimated HR for PO4 was consistent across analyses and sample size was limited for the prognostic factors, only the presence of liver metastases was retained in the final model for OS. However, the effect of prognostic factors will be further evaluated once phase 3 data becomes available, to address the current limitations of low sample sizes and the absence of a control arm.

Time-dependent serum PO4 concentration was a better biomarker of OS than average daily serum PO4 concentration up to 6 weeks (Table S3). However, HR estimates were similar for the two analyses (HR: 0.49 for longitudinal versus 0.57 previously). Lastly, serum PO4 until Day 14 (PO4_ave,2 weeks_) and erdafitinib free or total AUC were not better predictors of OS than average daily serum PO4 concentration (data not shown for early phosphate, Fig. 1C for free AUC). As an illustration, for total AUC, patients with higher AUC (highest tercile) showed a median OS of 6.5 months vs 9.7 months in patients with medium AUC (mid tercile) and 16.3 months in patients with lower AUC. Due to the inconsistent relationship with total AUC, further results are presented using free AUC only.

#### ER efficacy: PFS

Median PFS was 5.5 (95% CI 4.8, 5.6) months in the efficacy analysis dataset, with 15% of patients not having progressed or died at data cut-off. A total of 24 out of the 131 patients who showed disease progression (18%) remained on treatment beyond progression. When stratifying by dose regimen, median PFS was 5.5 months in the 8 mg versus 5.4 months in the 6 mg regimen. When stratifying by erdafitinib free AUC, patients with higher AUC (highest tercile) showed a median PFS of 5.7 months compared to 5.4 months in patients with medium AUC (mid tercile) and 5.2 months in patients with lower AUC (lowest tercile, Fig. 1D). Stratifying by serum PO4 (PO4_ave,6 weeks_) showed the greatest PFS differences. Patients with higher serum PO4 level (highest tercile) showed a median PFS of 5.6 months compared to 5.4 months in patients with medium PO4 level (mid tercile) and 4.3 months in patients with lower serum PO4 level (lowest tercile). The ER analysis revealed a statistically significant relationship between serum PO4 level and PFS, both when serum PO4 concentration was categorized by terciles (*p* = 0.044, Fig. 1B) and when it was used as a continuous variable (HR 0.84; 95% CI 0.70, 1.00 per 1 mg/dL increase in average daily serum PO4 concentration; *p* = 0.05; final model, Table 2).

When testing the effect of the prognostic factors in univariate analyses, the presence of high ECOG and liver metastases were found statistically significant adverse prognostic factors (Table 2). As observed with OS, only the presence of liver metastases remained statistically significant in the presence of PO4. As the estimated HR for PO4 was consistent across analyses and the sample size was limited for the prognostic factors, only the presence of liver metastases was retained in the final model for PFS. However, as with OS the effect of prognostic factors on PFS will be further evaluated once Phase 3 data becomes available, to address the current limitations of low sample sizes and the absence of a control arm.

Time-dependent serum PO4 concentration was a better biomarker of the PFS compared to average daily serum PO4 concentration up to 6 weeks (Table S3). HR estimates were similar between the two analyses (HR 0.67 for longitudinal versus 0.80 previously). Finally, serum PO4 until Day 14 (PO4_ave,2 weeks_) and erdafitinib free or total AUC were not better biomarkers of PFS than serum PO4 up to 6 weeks (data not shown for total AUC and early phosphate, Fig. 1D for free AUC).

#### ER efficacy: ORR

**Fig. 2:**
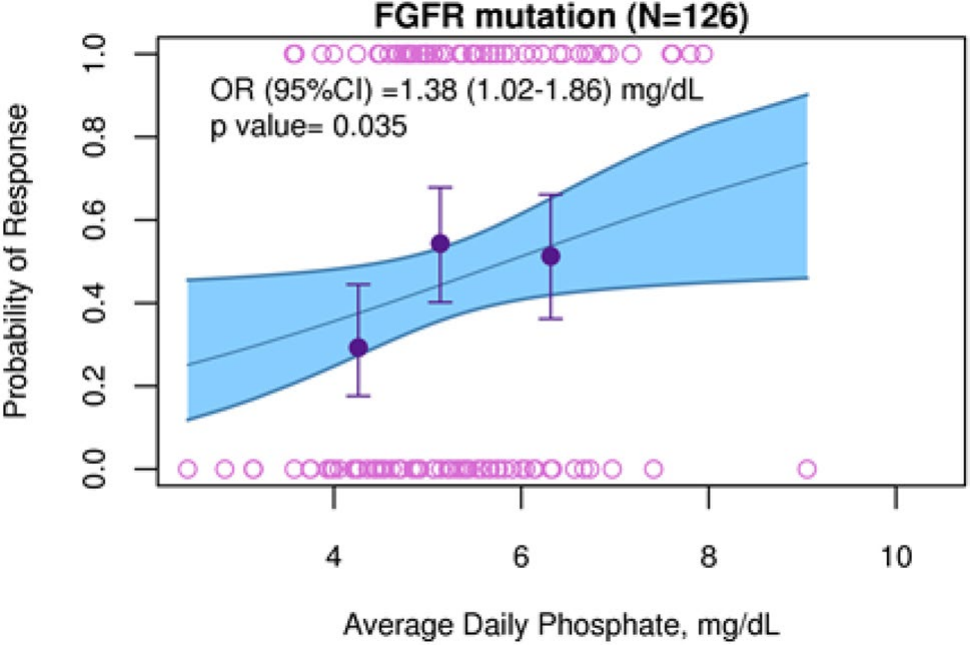
Probability of response as a function of average daily phosphate for FGFR mutations

The ORR of the efficacy analysis dataset was 40.4% (63/156; 95% CI 36.2%, 48.5%). Responses were generally achieved by the first disease assessment at 6 weeks. When stratifying by dose regimen, investigator-assessed ORR was 42.0% in the 8 mg versus 38.2% in the 6 mg regimen. When stratifying by free erdafitinib AUC (AUC_ave,6 weeks_), patients with higher AUC (highest tercile) showed an ORR of 43.4% compared to 45.3% in patients with medium AUC (mid tercile) and 32.0% in patients with lower AUC (lowest tercile). Stratifying by serum PO4 (PO4_ave`,6 weeks_) showed the greatest ORR differences. Patients with higher serum PO4 level (highest tercile) showed an ORR of 44.4% compared to 49.1% in patients with medium PO4 level (mid tercile) and 26.5% in patients with lower serum PO4 level (lowest tercile). The ER analysis for the ORR revealed a (borderline) statistically significant relationship between the serum PO4 level and ORR, both when serum PO4 concentration was categorized by terciles (*p* = 0.05) and when it was used as a continuous variable (*p* = 0.04; Table 2). A 1 mg/dL increase in average serum PO4 concentration was estimated to increase the odds of response by 1.38 (95% CI 1.02, 1.86; *p* = 0.04; Table 2, final model, and Fig. 2).

When testing the effect of the prognostic factors in univariate analyses, only the type of FGFR alteration was found a statistically significant prognostic factor (Table 2). Patients with FGFR fusions exhibited lower ORR than those with FGFR mutations (OR 0.26; 95% CI 0.10, 0.70; *p* = 0.01; Table 2 and Fig. S3). As observed for OS and PFS, serum PO4 until Day 14 (PO4_ave,2 weeks_) and erdafitinib AUC were not better biomarkers of ORR than average daily serum PO4 (data not shown).

### Exposure-safety analyses

The safety analysis dataset included 177 chemotherapy relapsed/refractory and chemotherapy naïve patients (*n* = 99 in 8 mg regimen; *n* = 78 in 6 mg regimen). The TEAEs with the highest incidence were nail disorders (50.8%) and eye disorders (50.3%), followed by skin disorders (35.6%), PPES (20.3%), and CSR (18.6%). Median time to event was 4.3 (95% CI 3.8, 5.0) months for nail disorders, 4.1 (95% CI 3.5, 6.4) months for eye disorders, and 7.7 (95% CI 6.0, NA) months for skin disorders. Median time to event was not available for CSR and PPES disorders as many patients did not experience an event at the time of analysis. For CSR and PPES disorders, the time for 25% of the events to have occurred was 6.6 (95% CI 4.0, 11.0) months and 7.4 (95% CI 3.6, NA) months, respectively.

**Fig. 3:**
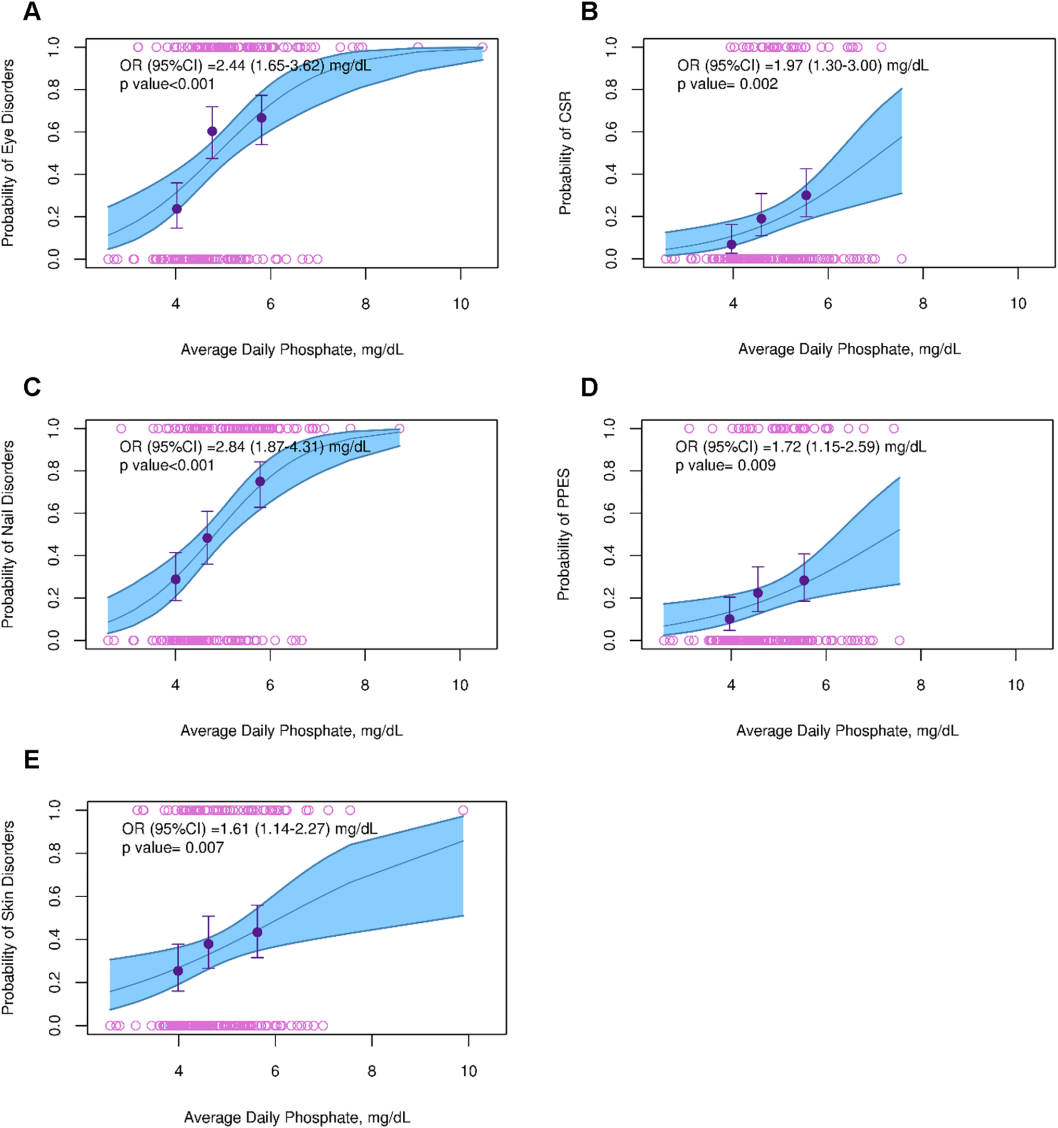
Probability of experiencing eye (**A**), CSR (**B**), nail (**C**), PPES (**D**), and skin disorders (**E**). The upper and lower open circles represent the presence or absence of disorder across the range of phosphate concentrations. The dots depict the observed incidence for the terciles of phosphate concentrations and the corresponding vertical bars represent the 95% CI. The full blue line and the associated shaded area represent the model-based exposure efficacy relationship and its 95% CI. *CSR* central serous retinopathy; *CI* confidence interval; *OR* odds ratio; *PPES* palmar-plantar erythrodysesthesia syndrome

When stratifying by dose regimen, AE incidence was generally higher in the 8 mg versus the 6 mg regimen, with a maximum incidence difference of 13.0% between the two regimens (for nail disorders; Table S4). When stratifying by erdafitinib free AUC (AUC_ave,event_), patients with higher AUC (highest tercile) showed an incidence increase of up to 26.0% (for nail disorders) compared to patients with lower AUC (lowest tercile), but this trend was not consistent across AEs (Table S4). Stratifying by serum PO4 (PO4_ave,event_) showed the greatest AE incidence differences. Patients with higher serum PO4 level (highest tercile) showed AE incidences up to 46.2% higher compared to patients with lower serum PO4 level (lowest tercile), and this trend was consistent across AEs (Table S4). The ER analysis revealed a statistically significant relationship between the serum PO4 level and most AEs, both when serum PO4 concentration was categorized by terciles (*p* < 0.05 for all but skin disorders [*p* = 0.11]) and when it was used as a continuous variable (*p* < 0.01 for all disorders; Table 2, Table S5 and Fig. 3). A 1 mg/dL increase in average serum PO4 concentration up to the day of the event increased the odds of nail, eye, skin, CSR, and PPES disorders by 1.6–3.0-fold. As observed for the efficacy endpoints, PO4 until Day 14 (PO4_ave,2 weeks_) and erdafitinib AUC were not better biomarkers of AE incidences than average daily serum PO4.

## Discussion

The results of the exploratory analyses presented here quantified the relationship between serum PO4 concentrations, reflective of both exposure and sensitivity to erdafitinib, and efficacy and safety endpoints, and established the scientific basis to justify the erdafitinib dosing paradigm, a pharmacodynamically guided individual dose titration, based on serum PO4 concentration early in the course of treatment (between day 14 and day 21). In this way, the clinical dose could be adjusted either up or down to maximize the erdafitinib benefit-risk profile for each individual patient. In this case, using PD-monitoring instead of classical PK therapeutic drug monitoring is relevant as PD correlates better with outcomes, displays more variability than PK as it also accounts for PK variability, and is easy to routinely perform in any laboratory.

The presented analyses showed that serum PO4 was an adequate biomarker of erdafitinib’s on-target effects, as serum PO4 was associated with the efficacy and FGFR-linked safety endpoints investigated. The exposure-efficacy analyses suggested that higher doses and/or higher patient sensitivity to erdafitinib leading to higher PO4 concentrations were associated with better OS, PFS, and ORR. In such case a dose up-titration could improve erdafitinib efficacy as evidenced in BCL2001 study [12]. The exposure-safety analyses suggested that higher doses and/or patients with a higher sensitivity to erdafitinib (exemplified by a more pronounced rise in serum PO4) had a higher likelihood of developing the selected AEs. In such case a dose reduction or interruption could improve erdafitinib tolerability and therapeutic index.

The results of a single-arm study supported the association between early PO4 level changes and clinical endpoints (ORR, PFS and OS). The ongoing active-controlled phase 3 study will enable to estimate the unbiased effect of drug-related PO4 level changes as a surrogate of erdafitinib effect, as phosphate will also be observed in a non-erdafitinib-treated population. Phosphate levels might be affected by the disease, in which case phosphate changes over time could reflect both drug exposure and progression of the disease. It is thus difficult to differentiate predictive from prognostic phosphate effects in this analysis. While this is certainly a limitation of the current analysis, we do not feel the results suggest a potential confounding. The more marked effect of erdafitinib on OS than PFS can be seen as evidence for phosphate to better reflect disease characteristics and not drug exposure. However, a number of arguments speak against this hypothesis: first, a similar behavior was recently presented for bemarituzumab, an anti-FGFR2b antibody [18]. Second, our study allowed treatment to continue post-progression, and around 18% of patients remained on study treatment after progression, which could have an impact on OS. Lastly, the use of early phosphate (first 6 weeks) and change from baseline phosphate more likely reflect changes due to drug exposure than disease progression.

Despite previous findings in the literature [19, 20], among investigated prognostic factors, only the presence of liver metastases was found significantly correlated with OS and PFS. This exploratory finding needs to be taken with great care given the limited sample size in the least frequent categories (12–35 patients). Given the robustness of the PO4-related HR across analyses and its superiority (in terms of AIC) over other prognostic factors, the final OS and PFS models did not include any prognostic factor except the presence of liver metastases. The final model for ORR included FGFR alterations, where fusions were a significant adverse prognostic factor in both univariate and multivariate analyses. Despite FGFR fusions patients displaying a lower ORR (CR + PR), many of them had prolonged stable disease. It is, therefore, conceivable that fusion is a significant covariate for ORR, but not for PFS nor OS, both of which are not dependent on just response but also are significantly influenced by durable (i.e. prolonged) stable disease. Note that phosphate-lowering drugs were not found to modify the phosphate effects on OS, PFS or ORR. In any case, the effect of prognostic factors, notably the correlation between hemoglobin and OS [19, 20], will be further evaluated once Phase 3 data becomes available, to address the current limitations of low sample sizes and the absence of a control arm.

The relationship between serum PO4 and OS/PFS was stronger than that between serum PO4 and ORR. A hypothesis for this is that many treated patients experience stable disease. These patients are considered non-responders, but they may nevertheless show long OS/PFS. The reason why the relationship is stronger with OS than with PFS remains unclear.

The ER analyses also supported that serum PO4 was a better biomarker for erdafitinib efficacy and safety outcomes than other exposure metrics, in particular PK metrics. As changes in serum PO4 concentration account for both the individual exposure to erdafitinib as well as the patient’s specific sensitivity to erdafitinib, this biomarker can be used for erdafitinib dose individualization. In the presented analyses, endpoints were correlated with average serum PO4 from the start of treatment until the first disease assessment (around 6 weeks) for efficacy and until the occurrence of AE for safety. A sensitivity analysis revealed that the absolute or relative changes in serum PO4 from each patient’s baseline did not show a better association with the different endpoints than the absolute PO4 value used in the primary analyses. This suggested that the defined threshold of 5.5 mg/dL (for up-titration) can be used regardless of individual PO4 baseline values. However, the investigated data contained PO4 values within a rather narrow range, which likely impaired the detection of potential differences between using relative and absolute changes. Additional data from studies allowing wider PO4 variations would be needed to confirm this hypothesis, notably from the ongoing phase 3 trial (THOR/BLC3001) where the PO4 threshold for dose adjustments was increased from 7 mg/dL in BLC2001 to 9 mg/dL in BLC3001.

In addition to the impact of baseline PO4, the impact of PO4 variations over time was also investigated. In the considered patient population, dosing was often interrupted and/or reduced due to AEs, irrespective of the serum PO4 level observed at that time, meaning that PO4 levels could fluctuate over time. Here, a weekly breakdown was considered relevant as most dose interruptions or changes happened on a weekly basis. To make the weekly analyses relevant, the period of PO4 prediction was extended from 6 weeks to the time of the event. For OS and PFS, weekly serum PO4 concentrations showed a better association than early PO4 concentrations, even if the estimated HR was similar between the two analyses. The investigation of the effect of PO4 over time on OS and PFS was, however, limited in this dataset due to the absence of a control arm, which would help disentangle the effect of time, PO4 and their interaction. The effect of longitudinal PO4 on OS and PFS will be further evaluated once data from the ongoing phase 3 controlled trial (THOR/BLC3001) become available.

The link between serum PO4 concentration and efficacy and safety endpoints as shown by the ER analyses can be used to support the approved dosing algorithm for erdafitinib, namely its starting dose of 8 mg, its potential pharmacodynamically guided individual up-titration to 9 mg between day 14 and day 21, and the proposed dose reduction by 1 or 2 mg at a time of AEs. Compared to the previous starting dose of 6 mg in Regimen 1 of the BLC2001 study, the current 8 mg dose (corresponding to a 0.56 mg/dL increase in average serum PO4) is predicted to decrease the hazard of death by 27% and the hazard of progression by 12%, while increasing the odds of response by 20% (Table 3)**.** The up-titration to 9 mg (corresponding to a 0.38 mg/dL increase in average serum PO4) is predicted to further improve the HR by 19% for OS, 8.1% for PFS and improve the OR by 13% for ORR (Table 3). Note that the reason for not starting at 9 mg erdafitinib upfront was based on individual tolerability as reported in the phase 1 study. Dose reduction by 1 or 2 mg as specified in the toxicity management guidelines is predicted to increase the tolerability of erdafitinib in patients that experience selected disorders. In fact, reducing the dose from 8 to 6 mg daily in patients who developed selected AEs was predicted to decrease the odds of eye disorders, CSR, nail disorders, PPES and skin disorders by 39%, 32%, 44%, 26% and 23%, respectively (Table 3). The presented efficacy findings also support limiting dose interruptions to those that are absolutely needed and limiting their duration to be able to restart treatment as soon as possible, as stated in the labeled dosing regimen.

**Table 3:** Predicted effects of dosing algorithm on efficacy and safety endpoints

Dose change of dosing algorithm	PK-PD predicted PO4 change	Effect OR/HR (95% CI)
Increase starting dose from 6 to 8 mg (regimen 1 vs. regimen 2)	+ 0.56 mg/dL	Decrease in HR of: 27% (95% CI 17, 35%) for OS 12% (95% CI 3.4, 20%) for PFS Increase in OR of: 20% (95% CI 1, 42%) for ORR
Up-titrate to 9 mg based on PO4 level at 2–3 weeks	+ 0.38 mg/dL	Decrease in HR of: 19% (95% CI 17, 26%) for OS 8.1% (95% CI 3.4, 14%) for PFS Increase in OR of: 13% (95% CI 0.8, 42%) for ORR
Reduce dose from 8 to 6 mg in case of AE	− 0.56 mg/dL	Decrease in OR (95% CI) of: 39% (24, 51%) for eye disorders 32% (14, 46%) for CSR 44% (30, 56%) for nail disorders 26% (7.5, 41%) for PPES 23% (7.1, 37%) for skin disorders

*CI* confidence interval; *CSR* central serous retinopathy; *HR* hazard ratio; *OR* odds ratio; *ORR* objective response rate; *PFS* progression-free survival; *PPES* palmar-plantar erythrodysaesthesia syndrome

This study was not without limitations. First, in addition to the issues identified for the evaluation of prognostic factors, the absence of a control arm did not enable (1) to identify the net erdafitinib effect on the endpoints evaluated, and (2) to differentiate between the effects associated with treatment from those related to time and disease progression. In this context, the results of the ER analyses only pertained to patients on erdafitinib treatment. The investigation of the effects of the attenuation of serum PO4 concentrations over time despite continuous erdafitinib exposure was exploratory and served to generate a hypothesis, which would need to be confirmed using additional data. While the limitation is an absence of a control arm, erdafitinib has a readily assessable surrogate for target engagement when compared to drugs with an alternate mechanism of action. The observed increase in serum PO4 is an encouraging sign of target engagement, which is hypothesized to translate into efficacy. This will need to be confirmed by the control arm of the ongoing Phase 3 trial.

A second limitation was that the serum PO4 biomarker found to be associated with the efficacy and safety endpoints evaluated in this analysis could not always be used in a prospective manner, especially when the average serum PO4 up to the time of the event was used. While serum PO4 at week 6 could be used to predict the likelihood of death and/or progression since these events typically occurred after multiple cycles of erdafitinib treatment, the average serum PO4 concentration up to the time of event could not be used to adjust the dose and maximize ORR or minimize AE incidence since the metric was only available at the time of the event of interest.

In conclusion, the ER analyses of efficacy and safety of erdafitinib suggest that higher serum PO4 concentration was associated with statistically significant improvement in OS, PFS and ORR in patients with mUC. Patients with higher serum PO4 concentration also had a statistically significantly increased incidence of nail disorders, eye disorders, skin disorders, PPES and CSR. These analyses supported the approved pharmacodynamically guided individual dose titration for the treatment of patients with mUC. Data generated in an ongoing randomized controlled clinical trial will be used to confirm the value of PO4 in erdafitinib dose-individualization.

## Supplementary Information

Below is the link to the electronic supplementary material.

Supplementary file1 (DOCX 485 KB)

## Data Availability

The data sharing policy of the study sponsor, Janssen Pharmaceutical Companies of Johnson & Johnson, is available at https://www.janssen.com/clinicaltrials/transparency. As noted on this site, requests for access to the study data can be submitted through Yale Open Data Access (YODA) Project site at http://yoda.yale.edu.
