# Supplementary material for: Exposure–response analyses of erdafitinib in patients with locally advanced or metastatic urothelial carcinoma
Source: Cancer Chemother Pharmacol. 2022 Jan 3;89(2):151–64. doi: 10.1007/s00280-021-04381-4 (PMC8807442; doi:10.1007/s00280-021-04381-4)
Supplement: Supplementary file 1 — Supplementary file1 (DOCX 485 KB) [file 280_2021_4381_MOESM1_ESM.docx]

**Supplement Appendix**

The safety endpoints were defined using the following terms:

- **Eye** (adverse event of clinical importance, AECI): Retinal thickening, Blepharitis, Cataract, Cataract subcapsular, Conjunctival hemorrhage, Conjunctival hyperemia, Conjunctival irritation, Corneal erosion, Corneal infiltrates, Dry eye, Eye inflammation, Eye irritation, Eye pain, Foreign body sensation in eyes, Keratitis, Lacrimation increased, Night blindness, Ocular hyperemia, Photophobia, Vision blurred, Visual acuity reduced, Visual impairment, Xanthopsia, Xerophthalmia, Chorioretinitis, Conjunctivitis.
- **CSR** (adverse drug reaction, ADR): Chorioretinopathy, Retinal detachment, Retinal oedema, Detachment of retinal pigment epithelium, Detachment of macular retinal pigment epithelium, Retinopathy, Vitreous detachment.
- **Nail** (AECI): Nail bed bleeding, Nail discoloration, Nail disorder, Nail dystrophy, Nail ridging, Nail disorder, Onychalgia, Onychoclasis, Onycholysis, Paronychia.
- **PPES** (ADR): Palmar-plantar erythrodysaesthesia syndrome, Palmar erythema.
- **Skin** (AECI): Dry skin, Skin fissures, Xeroderma, Skin exfoliation, Skin atrophy, Eczema, Eczema nummular, Hyperkeratosis, Pruritus, Skin lesion. Skin disorder.

**Efficacy endpoints**

OS was defined as the time between the date of the first dose of study drug and the date of the patient’s death, with patients alive censored at the time of the last visit. PFS was defined as the time between the date of the first dose of study drug and the date of disease progression, relapse, or death; censoring patients who have not progressed at the time of the last visit. ORR was defined as the proportion of patients with complete response (CR) or partial response (PR) based on the best objective response as determined by the investigator according to RECIST v1.1.

**Prognostic factors for exposure-efficacy analysis**

High ECOG performance status, low hemoglobin level, and the presence of liver metastases have been previously identified as the main adverse prognostic factors related to overall survival in patients with metastatic transitional cell carcinoma of the urothelial tract who experienced treatment failure with first‑line platinum‑based regimen.^1,2^ FGFR alteration type was included because it was used as randomization factor in BLC2001. Other prognostic or predictive factors such as demographics (e.g., age, sex, or race) were not considered, as none were found significant by statistical analysis. Phosphate-lowering comedication (such as denosumab, zoledronic acid and phosphate binders) were evaluated as an interaction term with serum phosphate to investigate whether their phosphate-lowering potential had an impact on drug effect.

**Statistical analysis**

The ER analyses were performed under the assumption that there was a sufficient number of events for a meaningful analysis. P-values <0.05 were considered statistically significant and no correction for multiple statistical testing was implemented. The nature of the current analysis was purely exploratory and considered a hypothesis generating exercise. Thus, the estimates of the parameters of interest, 95% CIs, and p values were determined to assist in evaluating the ER relationships, and therefore should be interpreted within the context of the models and the assumptions employed, as inherently done in model-based analysis methods.

The data management, graphical presentations, and the statistical analyses were performed using R Project for Statistical Computing, version 3.4.1 or higher for Windows. Derivation of the exposure metrics was done using NONMEM 7.3.

**Handling of missing data**

*Missing response (efficacy or safety) variable*

Missing observations or events were treated as missing and were not replaced with estimated (imputed) values. Patients with missing observation date were excluded from the analysis. Note that best response is not associated to a particular time, as it is derived from RECIST assessment under a set of conditions, including duration. For this analysis, best response was assigned to the time of response evaluation closest to the planned time of first evaluation, i.e. 6 weeks. This was considered reasonable given the expected very rapid onset of response to the drug.

*Missing prognostic and predictive factors*

If a given patient had a missing value in any prognostic or predictive factor, the patient was excluded from the analysis and no imputation was performed. If the number of patients with missing prognostic/predictive factor was relatively high (higher than 10%) a sensitivity analysis was conducted to compare erdafitinib effect on a given endpoint after assigning all the missing values to each of the possible efficacy prognostic/predictive factor categories.

*Missing exposure variable*

If the individual PK or PD parameter cannot be derived due to a lack of consistent or sufficient data, the typical values of PK and PD parameters were used to derive the corresponding exposure metrics. If the number of patients with missing exposure was relatively high (higher than 10%), a sensitivity analysis was conducted by excluding the patients with missing exposure.

*Exposure efficacy analysis*

According to a pre-specified analysis plan, the effect of serum PO4 and all prognostic factors were first tested in a univariate manner. Then, a multivariate model including serum PO4 and all prognostic factors was tested. Nonsignificant prognostic factors were then removed all at once from the full model, provided they had no confounding effect on serum PO4, i.e. provided that the OR/HR associated with serum PO4 did not substantially change. Bivariate models including PO4 and each univariately significant prognostic factors were also tested, and retained if statistically significant. Lastly, interactions were tested for significant prognostic factors which OR/HR differed between the univariate analysis and the full model. Only statistically significant interactions were retained.

**References:**

1. Bellmunt, J., Choueiri, T. K., Fougeray, R., Schutz, F. A., Salhi, Y., Winquist, E. *et al.* Prognostic factors in patients with advanced transitional cell carcinoma of the urothelial tract experiencing treatment failure with platinum-containing regimens. *J Clin Oncol* **28**, 1850-1855 (2010).
2. Dong, F., Shen, Y., Gao, F., Xu, T., Wang, X., Zhang, X. *et al.* Prognostic value of site-specific metastases and therapeutic roles of surgery for patients with metastatic bladder cancer: a population-based study. *Cancer Manag Res* **9**, 611-626 (2017).

**Figure S1: Prediction-corrected Visual Predictive Check of the PK Model Validation for Erdafitinib Plasma Concentrations**

Prediction-corrected concentrations are on the log scale. Red lines: median (solid line), 5^th^ and 95^th^ (dashed lines) percentiles of the simulated data with its 95% confidence intervals (blue and red shaded area); black lines: median (solid line), 5^th^ and 95^th^ (dashed lines) percentiles of the observed data for erdafitinib.

**Figure S2: Prediction-corrected Visual Predictive Check of the PK-PD Model Validation for Serum Phosphate Concentrations**

Red lines: median (solid line), 5^th^ and 95^th^ (dashed lines) percentiles of the simulated data with its 95% confidence intervals (blue and red shaded area); black lines: median (solid line), 5^th^ and 95^th^ (dashed lines) percentiles of the observed data for serum phosphate.

**Figure S3: Probability of Response as a Function of Average Daily Phosphate for FGFR Fusions**


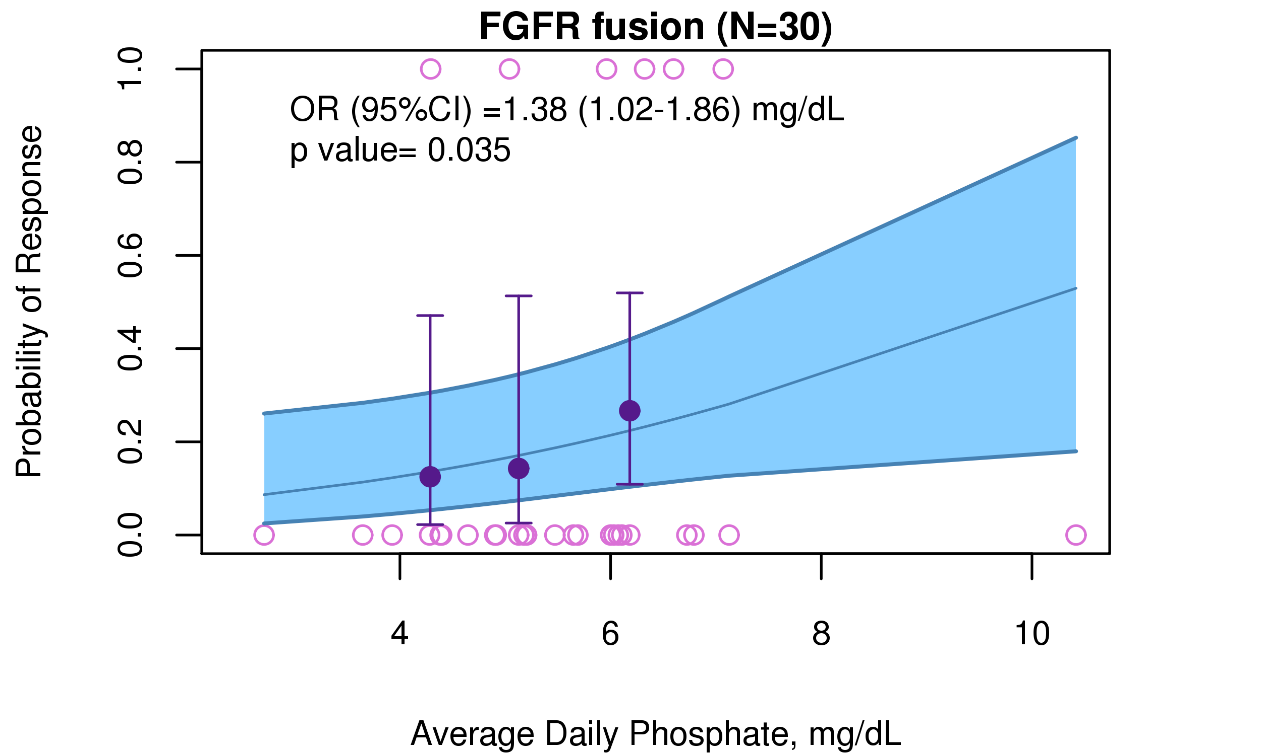


Note: Odds Ratio is for 1 mg/dL increase in average daily serum phosphate concentration; p-value are rounded to 2 decimals and have 0.001 as lower bound

**Table S1: Summary of demographic and disease characteristics of the patients at baseline in the efficacy exposure-response analysis dataset by phosphate tercile**

| **Variable** | **Labels** | **Low PO4**  **tercile** | **Middle PO4 tercile** | **High PO4**  **tercile** |
| --- | --- | --- | --- | --- |
| **N** |  | **49** | **53** | **54** |
| Disease distribution, n(%) | Presence of liver metastases | 20 (40.8) | 12 (22.6) | 8 (14.8) |
|  | Presence of bone metastases | 13 (26.5) | 7 (13.2) | 12 (22.2) |
|  | Presence of lung metastases | 26 (53.1) | 30 (56.6) | 30 (55.6) |
| FGFR alteration type, n (%) | Mutation | 41 (83.7) | 46 (86.8) | 39 (72.2) |
|  | Fusion | 8 (16.3) | 7 (13.2) | 15 (27.8) |
| ECOG, n (%) | ECOG 0 or 1 | 42 (85.7) | 51 (96.2) | 51 (94.4) |
|  | ECOG>1 | 7 (14.3) | 2 (3.8) | 3 (5.6) |
| Hemoglobin, n (%) | Hb >10 g/L | 33 (67.3) | 46 (86.8) | 42 (77.8) |
|  | Hb ≤10 g/L | 16 (32.7) | 7 (13.2) | 12 (22.2) |

ECOG, Eastern Cooperative Oncology Group; FGFR: fibroblast growth factor receptor; PO4, phosphate

**Table S2: Summary of mean ± SD (minimum-maximum) PK-PD model-predicted phosphate concentrations for the continuous regimens of study BLC2001**

| Analysis | Exposure metric | N | Pooled 6 and 8 mg Once Daily | 6 mg Once Daily | 8 mg Once Daily |
| --- | --- | --- | --- | --- | --- |
| Efficacy, phosphate | Average phosphate between Day 1 and Week 6 (mg/dL) | 156 | 5.30±1.16 (2.44-10.42) | 5.08±1.12 (2.44-9.05) | 5.47±1.16 (2.71-10.42) |
| Safety ^a^, phosphate | Average phosphate between Day 1 and Day of adverse event (mg/dL) | 177 | 4.96±1.13 (2.58-10.46) | 4.74±1.00  (2.58-9.10) | 5.13±1.21 (2.78-10.46) |

^a^All parameters are derived from the safety analysis of eye disorder as an example.

The p-values for the t-tests of differences in mean phosphate between dose groups 6 mg and 8 mg was p=0.03 for the efficacy data and p<0.001 for the safety data.

**Table S3: Results of OS and PFS analyses when the risk of progression/death depends on weekly phosphate**

| **Parameter** | **-2LL^a^** | **AIC^a^** | **HR (95% CI)^b^**  for 1 mg/dL increase in average weekly PO4 concentrations until treatment discontinuation | **p-value^c^** |
| --- | --- | --- | --- | --- |
| OS | 617.4 | 619.4 | 0.49 (0.41; 0.59) | <0.001 |
| PFS | 1011.5 | 1013.5 | 0.67 (0.62; 0.72) | <0.001 |

For these analyses, the effect of PO4 on OS and PFS was assumed to be zero after treatment with erdafitinib was discontinued.

-2LL: -2 log-likelihood; AIC: Akaike’s Information Criterion; CI: confidence interval; CSR: central serous retinopathy; HR: hazard ratio; OR: odds ratio; ORR: objective response rate; PFS: progression-free survival; PPES: palmar-plantar erythrodysaesthesia syndrome; PO4_ave,6weeks_ average daily serum phosphate until the first response assessment; PO4_ave,event_ average daily serum phosphate until the first highest grade adverse event

^a^ Best model has significantly lower -2LL (nested models) or lowest AIC (non-nested models)

^b^ HR for OS and PFS

^c^ p-value are rounded to 2 decimals and have 0.001 as lower bound

**Table S4: AE incidence by dose regimen, plasma free erdafitinib AUC and serum phosphate concentrations terciles**

| **AE** | **Dose regimen**  **N/Ntot (%)** | **Increasing erdafitinib free AUC_ave,event_ tercile**  **N/Ntot (%)** | **Increasing PO4_ave,event_ tercile**  **N/Ntot (%)** |
| --- | --- | --- | --- |
| Nail disorders |  | T1: 22/59 (37.3) | T1: 17/59 (28.8) |
|  | 6 mg: 34/78 (43.6)  8 mg: 56/99 (56.6) | T2: 30/58 (51.7) | T2: 28/58 (48.3) |
|  |  | T3: 38/60 (63.3) | T3: 45/60 (75.0) |
| Eye Disorders |  | T1: 25/59 (42.4) | T1: 14/59 (23.7) |
|  | 6 mg: 35/78 (44.9)  8 mg: 54/99 (54.5) | T2: 31/58 (53.4) | T2: 35/58 (60.3) |
|  |  | T3: 33/60 (55.0) | T3: 40/60 (66.7) |
| Skin disorders |  | T1: 24/59 (40.7) | T1: 15/59 (25.4) |
|  | 6 mg: 24/78 (30.8)  8 mg: 39/99 (39.4) | T2: 19/58 (32.8) | T2: 22/58 (37.9) |
|  |  | T3: 20/60 (33.3) | T3: 26/60 (43.3) |
| PPES |  | T1: 8/59 (13.6) | T1:6/59 (10.2) |
|  | 6 mg: 14/78 (17.9)  8 mg: 22/99 (22.2) | T2: 10/58 (17.2) | T2: 13/58 (22.4) |
|  |  | T3: 18/60 (30.0) | T3: 17/60 (28.3) |
| CSR |  | T1: 6/59 (10.2) | T1: 4/59 (6.8) |
|  | 6 mg: 11/78 (14.1)  8 mg: 22/99 (22.2) | T2: 17/58 (29.3) | T2: 11/58 (19.0) |
|  |  | T3: 10/60 (16.7) | T3: 18/60 (30.0) |

CSR: central serous retinopathy; PPES: palmar-plantar erythrodysesthesia syndrome.

Table S5. Univariate logistic regression analyses for treatment-emergent disorder as a function of free erdafitinib AUC

| **Disorder** | **Model** | **-2LL** | **AIC** | **OR (95% CI)** | **p-value** |
| --- | --- | --- | --- | --- | --- |
| Eye disorder | Average AUC_free,0-24_ up to day of event | 243.8 | 247.8 | 1.01 (1.00-1.01) | 0.218 |
| CSR | Average AUC_free,0-24_ up to day of event | 169.8 | 173.8 | 1.00 (0.99-1.01) | 0.476 |
| Nail disorder | Average AUC_free,0-24_ up to day of event | 242.0 | 246.0 | 1.01 (1.00-1.02) | 0.085 |
| PPES | Average AUC_free,0-24_ up to day of event | 176.5 | 180.5 | 1.01 (1.00-1.02) | 0.122 |
| Skin disorder | Average AUC_free,0-24_ up to day of event | 228.4 | 232.4 | 0.99 (0 98-1.00) | 0.169 |

AIC, Akaike’s Information Criterion; AUC, area under the curve; CSR: central serous retinopathy; PPES: palmar-plantar erythrodysesthesia syndrome; OR, odds ratio
